# Animal Experiments in Periodontal and Peri-Implant Research: Are There Any Changes?

**DOI:** 10.3390/dj7020046

**Published:** 2019-05-01

**Authors:** Noémie Staubli, Julia C. Schmidt, Carin A. Rinne, Sabrina L. Signer-Buset, Fabiola R. Rodriguez, Clemens Walter

**Affiliations:** 1 Department of Periodontology, Endodontology and Cariology, University Centre for Dental Medicine, University of Basel, Hebelstrasse 3, 4056 Basel, Switzerland; noemi.staubli@unibas.ch (N.S.); julia.schmidt@unibas.ch (J.C.S.); carin.rinne@unibas.ch (C.A.R.); 2 Private Dental Office Grimm Zahnärzte, Horgen, Schweiz, 8810 Basel, Switzerland; sbuset@gmx.ch; 3 Private Dental Office Dentilus AG, Forchstrasse 99, 8032 Zurich, Switzerland; FabRod@gmx-topmail.de

**Keywords:** animal experiments, bibliometrics, periodontal and peri-implant research, Journal of Periodontology, Journal of Clinical Periodontology

## Abstract

Animal experiments are a source of debate. This bibliometric study aims to identify published research in two representative dental journals: the Journal of Periodontology (JP) and the Journal of Clinical Periodontology (JCP). Two time points (1982/83 and 2012/13) covering 30 years were chosen. Articles describing data from animal experiments were identified and the data were extracted and compared between journals and time points. In 1982/83, 27 animal studies were published in JP and 17 in JCP. For 2012/13, 54 animal studies were considered in JP and 37 in JCP. The species examined were predominantly dogs (37%) in JCP and rats (61%) in JP in 1982/83. In 2012/13, rodents accounted for 85% in JP and for 54% in JCP. The number of animals used per study increased by a factor of 1.6–2.6. The diversity of geographic origin and articles from emerging countries increased over time. The number of animals examined per study and the publications describing these experiments seemed to have increased in the journals analyzed in the last decades.

## 1. Introduction

For ethical reasons, animal experiments are a source of an ongoing scientific and non-scientific debate [1,2,3,4]. Animal studies are applied in dental research, in particular for periodontal and dental implant experiments [5,6,7,8,9,10]. With the application of animal models in periodontology and implantology, the pathogenesis of oral diseases, including experimental periodontitis or—later on—the role of oral bacteria in bisphosphonate-associated necrosis of the jaw, was aimed to be explored [11,12]. Several therapeutic approaches, including enamel matrix proteins as an adjunct to periodontal surgery, were tested in various settings [13]. In recent years, animal experiments have been applied with respect to the performance of dental implants or the pathogenesis and therapy of peri-implant diseases [14].

Beside ethical considerations, questions regarding the applicability of results from animal experiments to humans has led to the application of and the ongoing need for the “Replace, Reduce, Refine” approach to animal experiments [3,15]. In this context, in Switzerland, the number of animals used for experiments decreased from approximately 2,000,000 in 1983 to 600,000 in 2013 [16]. Recently, awareness of the dental scientific community has increased, particularly with respect to periodontal aspects and dental implants [17,18,19,20,21]. In this research, a critical appraisal of studies using animals was applied. These issues affect risk of bias, including accuracy of treatment effect estimates, and quality of reporting, in particular reporting on success parameters for replication in human clinical trials or the methodological quality, i.e., power analysis and sample size calculation for animal studies. Finally, questions regarding the translation of animal research findings to human clinical trials were raised [17,18,19,20,21].

The aim of this bibliometric study was to explore changes in the number of published animal studies, average number of examined animals in the studies, and origin of the publications over the period from 1982/83 to 2012/13 in two representative journals: the Journal of Periodontology (JP) and Journal of Clinical Periodontology (JCP).

## 2. Materials and Methods

Ethical approval was not required for this kind of bibliometric research.

### 2.1. Focused Questions

(a) How many publications dealing with experiments on animals were published in the JP and JCP in the years 1982/83 and 2012/13?

(b) Was there a change regarding the average number of animals examined in the studies over the time period?

(c) With respect to articles with animal experiments, was there a difference regarding the animal species examined between these two periodontal journals?

(d) Where did the publications originate from?

### 2.2. Literature Search Strategy

#### Selected Journals and Time Frame

The search strategy and the methods applied were recently described in Reference [22]. Briefly, two leading highly ranked peer-reviewed periodontal journals with different geographical origin and representing the academic societies of North America and Europe, i.e., JP and JCP, were selected. The JP was ranked at position 9 with an impact factor (IF) 3.392 and the JCP as ranked at position 6 with an IF 4.046 in 2017 [23]. The search was developed to cover a reasonable time frame of 30 years (1982/83–2012/13). In the years 1982/83, a landmark paper on guided-tissue regeneration in animals (GTR) was published by Nyman [22,24].

### 2.3. Evaluation of Publications

For the focused question applied in this bibliometric research project, the following inclusion and exclusion criteria were applied:

#### 2.3.1. Inclusion Criteria and Applied Definition

Studies focused on animal experiments with all types of contents were considered in this analysis.

#### 2.3.2. Exclusion Criteria and Applied Definition

Publications assigned to “intervention studies in humans”, “epidemiological studies”, “in vitro studies (with material from humans)”, “reviews”, “case reports and series”, “comments, letters, editorials and errata”, and “others” were excluded. 

For classification of articles, three tree diagrams (TD-A, TD-B, and TD-C) were developed [22]. To evaluate the distribution of the origin of the published animal studies, the countries were allocated to seven groups: Europe, North America, South America, Australia, Asia, Africa, and Israel. The origin was deduced from the last author, since he or she represents the group leader and serves as principal investigator in most cases. A descriptive presentation of data using a frequency distribution was chosen [22,25].

## 3. Results

The initial agreement between the reviewers within the respective TD was 94.5% for the study design (TD-A). The data were presented according to a modification of the PICO-approach, i.e., population/participants (P), intervention (I), comparison (C), outcome (O) [26].

### 3.1. Population/Participants (P)—Number of Screened Articles and Authors

**Figure 1:**
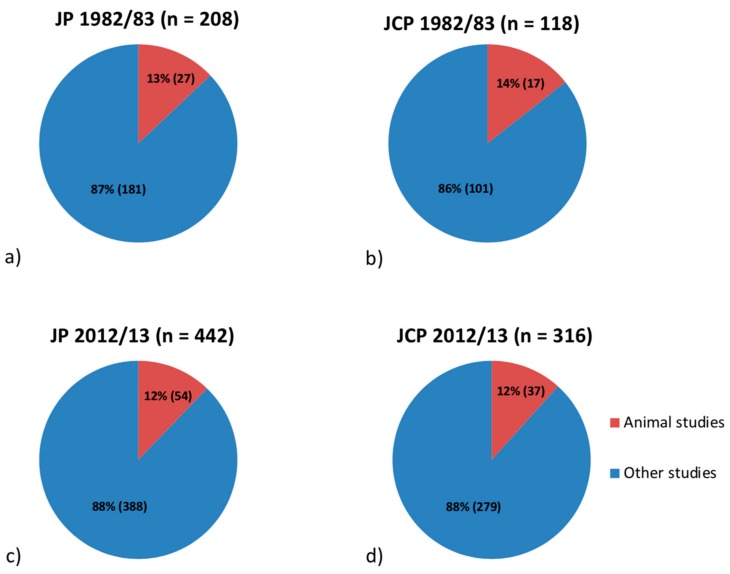
Proportion of articles with examinations on animals in the Journal of Periodontology (JP) (**a**) and the Journal of Clinical Periodontology (JCP) (**b**) in 1982/83, and in JP (**c**) and JCP (**d**) in 2012/13. Some articles were classified in more than one category, leading to discrepancies between the number of articles published, as is shown in the diagrams.

**Figure 2:**
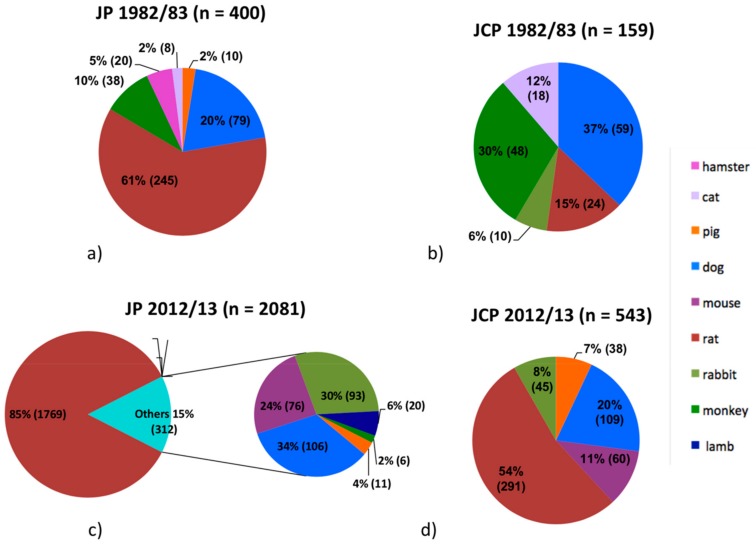
Amount and distribution of different animal species examined in articles published in JP (**a**) and JCP (**b**) in 1982/83, and in JP (**c**) and JCP (**d**) in 2012/13.

The data were presented according to journal and time point in Figure 1a–d and Figure 2a–d. Overall, 1084 articles were identified, and finally—after evaluation—135 articles describing animal experimentsconsidered.

The number of authors for each article depended on the year of publication and journal. A mean of 2.7 authors (JP) and 3.1 authors (JCP) contributed on average to an article in 1982/83. In contrast, on average, 6.8 authors (JP) and 7.1 authors (JCP) co-authored an article thirty years later.

### 3.2. Intervention (I)/Comparison (C)—Content of Screened Articles

In JP in 1982/83, out of 27 articles with animal experiments, 13 articles (48%) assessed “periodontal and peri-implant therapy”, 11 articles (41%) “etiology and pathogenesis”, five articles (19%) each “anatomy” or “others”, and one article (4%) “diagnostics”. In contrast, in JCP, out of 17 articles with animal experiments, most articles (9 articles/53%) assessed “etiology and pathogenesis”, followed by 6 articles (35%) assessing “periodontal and peri-implant therapy”, 5 articles (29%) “others”, and 3 articles (18%) “anatomy”. Thirty years later in JP, out of 54 articles, 27 articles (50%) examined “periodontal and peri-implant therapy”, followed by 26 articles (48%) with content on “etiology and pathogenesis”, 7 articles (13%) with “implant installation”, 4 articles (7%) each with “periodontal and peri-implant medicine” or “others”, 3 articles (6%) with “anatomy” and 1 article (2%) with “implant characteristics”. In JCP in 2012/13, out of a total of 37 animal experiments, the theme “etiology and pathogenesis” dominated with 15 articles (41%), followed by 14 articles (38%) with “periodontal and peri-implant therapy”, 6 articles (16%) with “implant characteristics”, 4 articles (11%) with “implant installation”, and 3 articles (8%) each with “anatomy” or “others”.

### 3.3. Outcome (O)

#### 3.3.1. How Many Publications Dealing with Experiments on Animals Were Published in the JP and JCP in the Years 1982/83 and 2012/13?

Articles with examinations on animals accounted for 27 articles (13%) in JP (Figure 1a) and 17 articles (14%) in JCP (Figure 1b) for the first time period, and 54 articles (12%) in JP (Figure 1c) and 37 articles (12%) in JCP (Figure 1d) for the second time period.

#### 3.3.2. Was there a Change Regarding the Average Number of Animals Examined in the Animal Studies over Time?

Overall, the 27 articles published in JP in 1982/83 examined 400 animals, leading to a mean of 14.8 animals per study (Figure 2a). In JCP, 159 animals were examined in 17 articles, with a mean of 9.4 animals per study (Figure 2b). Thirty years later, the overall number of animals examined accounted to 2081 animals out of 54 articles published in JP, leading to a mean of 38.5 animals per study (Figure 2c). In JCP, 543 animals were examined in 37 articles, with a mean of 14.7 animals per study (Figure 2d).

#### 3.3.3. Was there a Difference Regarding the Animal Species Examined between these Two Periodontal Journals?

In 1982/83, in JP, predominantly rats (245 rats, 61%) were examined, followed by 79 dogs (20%), 38 monkeys (10%), 20 hamsters (5%), 10 pigs (2%), and 8 cats (2%) (Figure 2a). In that time period in the JCP, predominantly dogs (59, 37%) and monkeys (48, 30%) were used (Figure 2b). The minority consisted of 24 rats (15%), 18 cats (12%), and 10 rabbits (6%). Thirty years later, most animals analyzed were rats and accounted for 85% in the JP and 54% in the JCP (Figure 2c,d). In JCP in 2012/13, additionally, 109 dogs (20%), 60 mice (11%), 45 rabbits (8%), and 38 pigs (7%) were examined.

#### 3.3.4. Where did the Publications Originate from?

In the first time period, the origin of the published literature behaved inversely. In JP, 81% of the articles (22 articles) originated from North America and 19% (5 articles) from Europe; in contrast, articles published in JCP originated more frequently from Europe (10 articles, 59%) than from North America (6 articles, 35%) and Australia (1 article, 6%). In 2012/13 in JP, most animal studies originated from Asia (25 articles, 46%) followed by South America (15 articles, 28%), North America and Europe (5 articles each, 9%), Israel (3 articles, 6%), and Africa (1 article, 2%). In the same time period in JCP, most articles originated from Asia and Europe (12 articles each, 32%) followed by South America and North America (5 articles each, 14%), and Israel (4 articles, 11%).

## 4. Discussion

This analysis revealed an increase in the absolute number of publications with examinations on animals in JP and JCP over 30 years of time. This number has doubled between the years 1982/83 until 2012/13. However, due to an increase in articles per journal issue, the percentage number of studies with examinations on animals out of all published articles has slightly decreased.

Animal experiments are an ongoing source of debate in science and public interest [1,2,3,4]. The theme received increasing attention in dental and peri-implant research and critical remarks were published. In several countries, but not in all, there is a trend for stronger regulation of animal experiments [27]. In light of this development driven from medical and pharmaceutical research, the question of this analysis was an evaluation of the number and content of publications on animal experiments in periodontal and peri-implant literature. For this purpose, two leading periodontal journals, i.e., JP and JCP, were selected, and a comparison within a reasonable time frame, i.e., 30 years, was conducted. However, it is likely that some animal studies, especially those in implantology, have shifted to other, more specialized journals in recent years.

The “Replace, Reduce, Refine” approach to animal experiments has a long tradition and was recently refreshed in a new position paper, i.e., the “Basel Declaration” [2,3]. The concept of the three R’s was first proposed in the textbook “The Principles of Humane Experimental Technique” by Russell and Burch in 1959 [28]. It has become a widely accepted milestone on ethical principles [29].

In 1982/83, most studies originated from North America and Europe. In 2012/13, a greater diversity with respect to origin of study was observed, i.e., Asia published the most articles on animal studies. This publication trend may reflect the economic and scientific development of countries from emerging markets, i.e., the BRIC countries (Brazil, Russia, India, China) and Turkey [30,31]. However, this trend may indicate some differences with respect to legislation and ethical considerations with animal experiments [30].

In the present analysis, articles on animal experiments published in JP or JCP originating from North America (28 articles in 1982/83, 10 articles in 2012/13) decreased in the observed time period, while animal studies originating from Europe (15 articles in 1982/83, 17 articles in 2012/13) remained constant in these journals. In studies originating from North America, a reduction from 424 animals in 1982/83 to 204 animals in 2012/13 occurred compared to studies originating from Europe, with data from 111 animals used in 1982/83 and 309 animals used in 2012/13. However, the awareness of animal experiments is not homogeneous in Europe. While, in Switzerland, the number of animals used for experiments decreased from approximately 2,000,000 animals in 1983 to 600,000 animals in 2013 [16], in the United Kingdom, an ongoing increase was recently stated [27]. Specifically, in the field of cosmetics, the European Union (EU), India, Israel, and Norway worked to ban cosmetics testing on animals [32]. Recently, China became part of this development and released requirements on animal testing for cosmetics. In the past, all cosmetics sold in China went to animal testing before any active marketing began [33,34].

The mean number of animals examined in a study increased from 1982/83 to 2012/13, and the distribution of animal species used changed considerably. The percentage number of examined dogs and monkeys has decreased over time, with rats being predominantly used in 2012/13 rats (Figure 2a–d). In the regulations for scientific procedures with animals, founded by member states of the EU in 1986, species including horses, dogs, cats, and non-human primates are specially protected [29]. This may be the reason for the decrease of these animal species between 1982/83 and 2012/13. Today, laboratory animals, e.g., rats, are commonly used for animal experiments. A reason for this might be that breeding rats is faster and cheaper than breeding monkeys or dogs. However, in the history of development, rodents are far away from humans, in contrast to monkeys.

This analysis revealed that a majority of articles with animal experiment content examined the pathogenesis and/or therapy of periodontal and peri-implant diseases. Obviously, the 1980s were dominated by studies with content more related to periodontology, while in the decade used for comparison, many studies were done on implants. However, the pathogenesis of human periodontitis and peri-implantitis is considered as a multifactorial system [35,36]. The understanding and the complexity increased. Recently, a new chart of the proposed pathogenesis was published [36]. According to this development, the transferability from animal research to understanding pathogenesis and/or therapy of human periodontal and/or peri-implant diseases remains a critical task or needs to be considered as an increasing challenge. Recently, an evaluation from mouse models showed “that although acute inflammatory stresses from different etiologies result in highly similar genomic responses in human, the responses in corresponding mouse models correlate poorly with the human conditions” [15]. An evaluation of the same database showed controversial results [37]. In addition to these drawbacks derived from timely genetic analyses, most chronic diseases, including periodontal diseases, are complex multifactorial diseases with increasing prevalence in aging populations. An example: for periodontal diseases some risk factors are established [35,36]. Onset and progression are caused by an opportunistic infection with a pathogenic oral biofilm. The composition of this biofilm shows a high intra-/inter-individually diversity and is affected by several behavioral factors, including nutrition,tobacco use and/or local environmental factors, including quality, extent, and material of fixed or removable dental prosthesis [38,39]. Moreover, evidence indicates education and lower socio-economic status as risk factors for periodontal diseases [40]. Most of these factors accumulate in the aging individual, explaining the higher prevalence in aging populations. Many of the characteristics listed above are not possible to adequately simulate in animal models on periodontal diseases.

Therefore, animal experiments do not provide direct evidence relevant for human periodontal or peri-implant diseases. To do so, they need to be replicated within human clinical trials [17]. The benefit for understanding pathogenesis or directions for therapy of human diseases needs to be critically defined for each experiment and for each animal used. In addition, to reduce risk of bias derived from animal experiments, a high precision of reporting of relevant data and a close adaption to quality guidelines such as the ARRIVE (animal research: reporting in vivo experiments) guidline [41] is required [18,20].

## 5. Conclusions

This bibliometric analysis indicated an increase of animal-associated publications in two leading periodontal journals. With respect to ethical considerations and the body of recent evidence for the scientific limits for transfer from research data derived from animals to pathogenesis and therapy of human diseases, researchers, editors, and publishers from our field are called:

(1) to ask a clear and precise research question with respect to the expected benefit for the therapy of human diseases;
(2) to focus on a strong adherence to the 3-R’s strategy, i.e., to replace, reduce, and refine animal experiments;
(3) to apply a high standard of reporting and analysis of data;
(4) to adhere to quality guidelines such as ARRIVE; and
(5) to critically review submitted manuscripts with respect to points 1, 2, 3, and 4.

## References

[1] NZZ (2009). Brandanschlag auf Vasellas Tiroler Jagdsitz. https://www.nzz.ch/vasella_brand_jagdsitz_tirol-1.3272074.

[2] Abbott A. (2010). Basel Declaration defends animal research. Nature.

[3] Basel Declaration (2011). A Call for More Trust, Transparency and Communication on Animal Research. http://www.basel-declaration.org.

[4] Cruelty Free International (2017). Facts and Figures on Animal Testing. https://www.crueltyfreeinternational.org/why-we-do-it/facts-and-figures-animal-testing.

[5] Egelberg J. (1966). Permeability of the dento-gingival blood vessels. 3. Chronically inflamed gingivae. J. Periodontal Res..

[6] Egelberg J. (1966). Permeability of the dento-gingival blood vessels. II. Clinically healthy gingivae. J. Periodontal Res..

[7] Egelberg J. (1966). Permeability of the dento-gingival blood vessels. 1. Application of the vascular labelling method and gingival fluid measurements. J. Periodontal Res..

[8] Egelberg J. (1966). The blood vessels of the dento-gingival junction. J. Periodontal Res..

[9] Perlstein M.I., Bissada N.F. (1977). Influence of Obesity and Hypertension on Severity of Periodontitis in Rats. Oral Surg. Oral Med. Oral Pathol. Oral Radiol. Endod..

[10] Brånemark P.I. (1983). Osseointegration and its experimental background. J. Prosthet. Dent..

[11] Hamp S.E., Lindhe J., Loe H. (1972). Experimental periodontitis in the beagle dog. J. Periodontal Res..

[12] Mawardi H., Giro G., Kajiya M., Ohta K., Almazrooa S., Alshwaimi E., Woo S.B., Nishimura I., Kawai T. (2011). A role of oral bacteria in bisphosphonate-induced osteonecrosis of the jaw. J. Dent. Res..

[13] Hammarström L., Heijl L., Gestrelius S. (1997). Periodontal regeneration in a buccal dehiscence model in monkeys after application of enamel matrix proteins. J. Clin. Periodontol..

[14] Ericsson I., Persson L.G., Berglundh T., Edlund T., Lindhe J. (1996). The effect of antimicrobial therapy on periimplantitis lesions. An experimental study in the dog. Clin. Oral Implant. Res..

[15] Seok J., Warren H.S., Cuenca A.G., Mindrinos M.N., Baker H.V., Xu W., Richards D.R., McDonald-Smith G.P., Gao H., Hennessy L. (2013). Genomic responses in mouse models poorly mimic human inflammatory diseases. Proc. Natl. Acad. Sci. USA.

[16] Bundesamt für Veterinärwesen BVET (2016). Anzahl Tiere von 1983–2016. http://tv-statistik.ch/de/statistik/index.php#a1.

[17] Faggion C.M., Schmitter M., Tu Y.K. (2009). Assessment of replication of research evidence from animals to humans in studies on peri-implantitis therapy. J. Dent..

[18] Faggion C.M., Giannakopoulos N.N., Listl S. (2011). Risk of bias of animal studies on regenerative procedures for periodontal and peri-implant bone defects—A systematic review. J. Clin. Periodontol..

[19] Faggion C.M., Listl S., Giannakopoulos N.N. (2012). The methodological quality of systematic reviews of animal studies in dentistry. Vet. J..

[20] Faggion C.M., Aranda L., Diaz K.T., Shih M.C., Tu Y.K., Alarcon M.A. (2016). The Quality of Reporting of Measures of Precision in Animal Experiments in Implant Dentistry: A Methodological Study. Int. J. Oral Maxillofac. Implant..

[21] Faggion C.M., Diaz K.T., Aranda L., Gabel F., Listl S., Alarcon M.A. (2017). The risk of bias of animal experiments in implant dentistry: A methodological study. Clin. Oral Implants Res..

[22] Staubli N., Schmidt J.C., Buset S.L., Gutekunst C.J., Rodriguez F.R., Schmidlin P.R., Walter C. (2018). Traditional or regenerative periodontal surgery?—A comparison of the publications between two periodontal journals over time. Clin. Oral Investig..

[23] JCR Science Edition (2016). Journals with Impact Factors on Dentistry, Oral Surgery & Medicine. https://www.google.ch/url?sa=t&rct=j&q=&esrc=s&source=web&cd=1&ved=0ahUKEwiamaPS-dXRAhWrAsAKHQ2ICl8QFggaMAA&url=https%3A%2F%2Flib.hku.hk%2Fsites%2Fall%2Ffiles%2Ffiles%2Fdenlib%2Fimpact%2520factor%25202015.pdf&usg=AFQjCNF1ow8h7gTqnLNhz53Icx5R5nf4MQ.

[24] Nyman S., Gottlow J., Karring T., Lindhe J. (1982). The regenerative potential of the periodontal ligament. An experimental study in the monkey. J. Clin. Periodontol..

[25] Park J.B., Ko Y., Park Y.G. (2015). Letters to the editor: Re: Bibliometrics study on authorship trends in periodontal literature from 1995 to 2010. J. Periodontol..

[26] Miller S.A., Forrest J.L. (2001). Enhancing your practice through evidence-based decision making: PICO, learning how to ask good questions. J. Evid. Based Dent. Pract..

[27] Siddique H. (2014). Number of Animal Experiments Continues to Rise in UK. https://www.theguardian.com/science/2014/jul/10/animal-experiments-rise-again-uk-genetic-research.

[28] Russell W.M.S., Burch R.L. (1959). The Principles of Humane Experimental Technique.

[29] Home Office (2017). User Guide to Annual Statistics of Scientific Procedures on Living Animals Great Britain. https://www.gov.uk/government/uploads/system/uploads/attachment_data/file/626965/guide-animal-procedures-commentary.pdf.

[30] Reichman J.H. (2009). Intellectual Property in the Twenty-First Century: Will the Developing Countries Lead or Follow?. Houst. Law Rev..

[31] Geminiani A., Ercoli C., Feng C., Caton J.G. (2014). Bibliometrics study on authorship trends in periodontal literature from 1995 to 2010. J. Periodontol..

[32] Kretzer M. (2015). Countries Around the World Work to Ban Cosmetics Testing on Animals. https://www.peta.org/blog/countries-around-the-world-work-to-ban-cosmetics-testing-on-animals/.

[33] European Commission (2013). Full EU Ban on Animal Testing for Cosmetics Enters into Force. http://europa.eu/rapid/press-release_IP-13-210_en.htm.

[34] Spencer N. (2017). PETA: China’s New Regulation Removes Animal Testing Procedure. https://www.cosmeticsdesign-asia.com/Article/2017/01/24/PETA-China-s-new-regulation-removes-animal-testing-procedure.

[35] Page R.C., Kornman K.S. (1997). The pathogenesis of human periodontitis: An introduction. Periodontology 2000.

[36] Meyle J., Chapple I. (2015). Molecular aspects of the pathogenesis of periodontitis. Periodontology 2000.

[37] Takao K., Miyakawa T. (2015). Genomic responses in mouse models greatly mimic human inflammatory diseases. Proc. Natl. Acad. Sci. USA.

[38] Amiri-Jezeh M., Rateitschak E., Weiger R., Walter C. (2006). The impact of the margin of restorations on periodontal health—A review. Schweiz. Mon. Für Zahnmed..

[39] Ramseier C.A., Warnakulasuriya S., Needleman I.G., Gallagher J.E., Lahtinen A., Ainamo A., Alajbeg I., Albert D., Al-Hazmi N., Antohé M.E. (2010). Consensus Report: 2nd European Workshop on Tabacco Use Prevention and Cessation for Oral Health Professionals. Int. Dent. J..

[40] Rodriguez F.R., Paganoni N., Weiger R., Walter C. (2017). Lower Educational Level is a Risk Factor for Tooth Loss—Analysis of a Swiss Population (KREBS Project). Oral Health Prev. Dent..

[41] Kilkenny C., Browne W., Cuthill I.C., Emerson M., Altman D.G. (2010). Animal research: Reporting in vivo experiments: The ARRIVE guidelines. Br. J. Pharmacol..

